# Chorea of the Soft Palate Caused by the Hypertrophy and Hyperæsthesia of the Mucous Membrane Covering Both Inferior Turbinated Bodies

**Published:** 1888-10

**Authors:** J. E. Schadle

**Affiliations:** St. Paul, Minnesota


### CHOREA OF THE SOFT PALATE CAUSED BY THE HYPERTROPHY AND HYPERÆSTHESIA OF THE MUCOUS MEMBRANE COVERING BOTH INFERIOR TURBINATED BODIES.*

BY J. E. SCHADLE, M. D., ST. PAUL, MINNESOTA.

That the origin of not a few reflex nervous disorders can, with a certain degree of exactness, be traced to the presence of morbid conditions of the naso-pharyngeal cavities, is a question no longer doubted.

The observations of recent writers on rhinological subjects furnish us conclusive evidence that some forms of headache and of spasmodic asthma are in many instances the outgrowth of a nasal polypus, a turbinated thickening, or a septal deformity.

*Read before the American Rhinological Association, Sept. 12, 1888.

The fact, too, is known that these reflex neurotic disturbances disappear soon after appropriate measures of treatment have been directed towards the removal of their cause.

The purpose of this paper is to call the attention of the honorable fellows of this Association to a case of choreiform movements of the soft palate appearing in a young lady whose family history is somewhat unusual as well as remarkable, and whose affliction is without doubt phenomenal.

The patient was referred to me by my friend, Dr. Baker, of this city, whose letter of introduction reads as follows:

ST. PAUL, MINN., April 16, 1888.

*Dear Doctor*—This interesting case came under my care June 3d, 1887. The only thing complained of was a constant contraction and relaxation of the levatores palati. Each contraction carried the uvula upward and backward till it came in contact momentarily with the upper and back part of the pharynx. The relaxation and separation of the moist mucous membrane surfaces caused the sound. The noise was like that produced by a rapidly ticking watch, and of the same frequency. It was never absent except during sleep. Having seen chorea in a girl of fourteen, during my early practice, in whom there was a constant irritative cough or effort to clear the throat, I recognized the present case as similar, but involving a different set of muscles. The young lady was in excellent health, all the bodily functions being well performed. My treatment was as follows:

℞—Zinci bromidi............................ʒi.

Syr. simplicis.........................f. ℥i.

M. Sig.—Ten drops in water four times a day; increase one drop each day until the dose nauseated.

This not producing the desired result, I gave tr. physostigmatis in doses ranging from fifteen to thirty drops. After continuing this remedy for a time, I abandoned it and prescribed fl. ext. cimicifugæ rac. in doses of f. ʒ ss. to f. ʒ i. every four hours.

About July 7th, 1887, I began the trial of galvanism. Used eight cells of battery, and placed the positive pole with a sponge electrode at the back of the neck; the negative pole, with a metal-tipped electrode such as is used for the urethra, was applied to the palate just above the uvula. The sittings were of five to ten minutes' duration, and I increased the number of cells to eleven. At the second treatment movements ceased and did not return for several hours. After the fifth and sixth application the ticking stopped and did not return till the first of this month when she called on me. Examinations showed enlarged tonsils with evidences of throat and nose disease. Thinking the condition of the nose and the throat caused a return of the choreic movements, and cure of same might permanently relieve, I now introduce her to you for examination and treatment. A singular feature of the case is that her general health had been almost perfect through all these years. Another item of note is that no other sets of muscles were involved. In the case before referred to her health was by no means good, and the choreic spasms became general, although a cure was obtained after long-continued treatment.

Yours truly, J. F. BAKER.

In the archives of Otology, Vol. xii, No. 1, March, 1883, this same case is reported by Dr. Cornelius Williams, a prominent oculist and aurist of this city. In this article Dr. Williams says: "When Violetta was ten years old, having occasion to get up during the night, she lost her way in going back to bed, and reaching her grandmother's room by mistake, she laid her hands upon the aged lady in the dark, and so alarmed her, and was herself so much frightened by the grandmother's shrieks, that she almost went into convulsions. She refused to return to her own bed, but lay in her sister's arms, starting and sobbing, the night through. Next day she was extremely pale and nervous, nor did she recover her wonted spirits for a number of days. This happening in the spring of 1880, and a short time after this the child discovered that a strange clicking sound was produced in her mouth, but suffering no inconvenience from it, she mentioned it to no one. In the June following she fell into Lake Elmo (Minn.) and came near being drowned, and a short time after this she called the attention of her mother to the clicking, which had now become constant. The family medical attendant was consulted, who pronounced it a common affair; the uvula was cut off entirely, and one tonsil was amputated without result as far as concerned the clicking. The patient is in good general health; appetite and digestion good; sleeps well. She is easily fatigued, but is kept up by any excitement. There has been for the last three years diurnal incontinence of urine, the act of micturition recurring about every half hour, but at night it is hardly ever necessary for her to get up more than twice. Dr. D. W. Hand, who was kind enough to examine her, informs me that there is considerable leucorrhœa, and that the urethra is unusually large and patulous. He explored the bladder and found no evidence of stone. The act of micturition is not painful. Drs. Hand, Boardman, Abbott and Wheaton examined her heart at my request, and report that there is nothing abnormal about it. Upon looking into the patient's mouth, it is perceived that the velum palati is rapidly raised and lowered without being made tense in its entirety. At the moment of relaxation of the levatores a sound is produced which is as much as can be like the ticking of a small brass clock, and in a still room it may be heard at a distance of twenty feet. The clicking corresponds to a complete contraction and relaxation of the levatores palati, and by actual count is 120 a minute, with very little variation in frequency at any time. When the mouth is opened widely the azygos uvulæ is sometimes seen to contract, but such contraction would seem to be physiological. The tone of the clicking is changed by closing the nose and by otherwise altering the usual conditions of the mouth and nose as to the volume of air contained; but that nor any other manipulation procures the cessation of the noise or its cause. Laryngoscopic examination shows the larynx to be normal, save a slight congestion. Rhinoscopy is not practicable. Otoscopy reveals the membrana tympani of each ear slightly indrawn, the handle and short process of the malleus of the right being abnormally prominent; light spot gone from both Mtt.

"By means of the diagnostic tube I am able to hear the clicking sound in either of the patient's ears—more distinctly in the right. It may very well be likened to the ticking of a watch under a pillow or the sound of the fœtal heart. If there is any movement of the membrana tympani I have not been so fortunate as to observe it. The girl's voice is natural, and she can sing with correctness, uttering the chest-notes without difficulty, but is unable to produce head-notes at all. In running the scale a decided tremolo is remarked. The patient, of necessity, breathes through the mouth, and from habit keeps it open during sleep. When there is tonsilitis, to which she is subject, there is considerable druling. At such times she is apt to have glottic spasm. The spasm of the levatores ceases during sleep. At irregular intervals, perhaps fifty or a hundred times through the day, there is an interrupted spasm of the diaphragm, giving rise to a sudden and deep inspiration in two or three motions, as in sobbing, followed by prolonged expiration. At times, it may be for an hour or half a day, she hears in her ears a sound comparable to the rapid revolution of a small fan-wheel. Acuteness of hearing, normal."

With the above information relating to the previous history, we are brought to the present consideration of this singular malady, a condition which I prefer to speak of as choreiform movements of the soft palate.

In company with her mother, Miss V. Z. presented herself at my office for examination on the 16th of April, 1888. She is a decided brunette, seventeen years of age. General appearance is good; tall and well developed; seems to be of a cheerful temperament.

Menstruation commenced normally, the epochs occurring regularly up to the present time.

Digestive organs in a fair condition; suffers from constipation and flatulency quite frequently. Urinary function, normal. Heart's action more or less excited; the heart beats tumultuously when ascending a flight of stairs, a hill or some other elevation.

Complains in a marked degree of obstructed nose-breathing.

Organization is of the nervous type; excitement affecting nervous system is not well borne.

Voice is impaired, especially when attempting head-notes, as in singing.

The principal affection complained of, and for which she was referred to me, is the spasmodic movement of the velum palati.

Of the family history, particularly that part of it relating to the father's side, much can be said which not only has a special bearing on the case under consideration, but also gives valuable information for the neurologist.

A predisposition to melancholia and suicide, handed down from the grandfather to the present generation, seems to exist.

The grandfather, a Hungarian by birth and a school teacher by occupation, shot himself at the age of fifty-eight.

An aunt, fifty-five years old, drowned herself in 1885.

She was married and mother of five children, one of whom, a son, aged twenty-eight years, came to his death two years ago by shooting himself. Another son, still living, suffered from melancholia for sometime after the occurrence of the tragic deaths of mother and brother.

Conrad, father of Miss Z., at the age of forty-six, drowned himself while suffering from a fit of insanity. It is said he was an intelligent gentleman and an accomplished musician.

In July, this year, a brother of Miss Z., aged twenty-eight, died from the effects of carbolic acid, which he drank with a view to self-destruction, this being his second attempt at suicide.

The mother is healthy. Aside from an ancestral strumous tendency, her family record is good.

A faucial examination reveals distinct rhythmical choreiform movements of the velum palati, accompanied by a peculiar clicking sound, distinctly audible for a distance of twelve or fifteen feet from the patient. The levators of the palate seem to be the ones especially engaged in the production of these involuntary muscular phenomena. The levatores act in unison with each other and volition has no control over their behavior. The mucous membrane covering the palate and adjacent structures is anæmic, and has adhering to it a glistening, tenacious secretion which, by ordinary attempts, is difficult to remove. The left palatal tonsil is larger than it normally ought to be, a condition due to frequent attacks of acute tonsilitis, to which she has been subject since childhood. Only a remnant of the uvula is to be seen. By an anterior rhinoscopic examination, I find chronic hypertrophy of the inferior turbinated bones, and, in a more marked degree, of the middle ones, the presence of whose redundant tissue exerts pressure on the septum and produces obstruction of the middle meatus. Posterior examination with the rhinoscope brings into view unusual chronic enlargement of the pharyngeal tonsil. It is lobular and irregular in shape and extends, with its free extremity, from a broad base in the vault to some distance below the upper margins of the canal of the nose. Inspection of the posterior nares presents excessive chronic thickening of the pituitary membrane covering the posterior third of the inferior turbinated bones. That part of the nasal passage is almost occluded by this condition. The aspect of the septum posteriorly is thin and pale.

There is an important feature entering into the history of this case which must not be overlooked. I refer to the extremely hyperæsthetic state of the intra-nasal mucous membrane. Mackenzie's sensitive areas are prominently characterized by it. Even the margins of the nostrils sympathize in this respect with the mucous membrane lying beyond them. The presence of a speculum causes a disagreeable feeling and creates nervous irritation. The use of the probe in the chambers of the nose is utterly intolerable; contact of it with any portion of the membrane, and more particularly with the posterior part of the inferior turbinated bones, at once gives rise to violent sneezing and spasmodic coughing. A flow of tears invariably follows these manipulations.

TREATMENT.—My treatment was mainly surgical. The first impression entertained was that possibly the enlarged pharyngeal tonsil might have been a source of the trouble, and that a thorough removal of it would do away with a foreign element constantly coming in contact with the opposing surface of the velum palati, and exciting thus into action clonic spasms. Snaring was impracticable.

I therefore, by the use of Cohen's post-nasal cutting forceps, and of the electro-cautery, reduced the growth in its entirety. This procedure was ensued by cessation of the choreiform movements, which remained absent for a period of two weeks, when they suddenly returned during a spell of nervous excitement, from which the young lady was suffering.

My attention was now turned to the treatment of the nasal passages.

The pathological changes found in them offered a theory for a reflex cause of the affection. Cocainizing the intra-nasal mucous membrane generally produced relief for half an hour. With a view to reduce the hyperæsthesia and hypertrophy of the turbinated bodies, I employed the electro-cautery, thoroughly burning the affected parts. An immediate result took place; the trouble at once ceased. Two cauterizations were necessary to affect entire perviousness of the nostrils and overcome obstructed respiration of the nose. Nose-breathing displaced mouth-breathing, and the dry sensation and sticky secretions of the pharynx gradually disappeared. The patient soon showed improvement in other respects.

The functional disturbances of the heart and shortness of breath passed off, while the general nervous system also gained tone. She also can now speak and sing with a clearer voice, the head-tones improving daily.

Beyond a doubt, a cure has been produced, the permanency of which already having been severely tested.

The melancholy death of her brother by suicide, mentioned in the family history, having occurred in the meantime, in itself was sufficient to reproduce the disturbance in consequence of intense emotional excitement into which she was thrown at the time. Upon receiving the news she immediately was seized by a violent attack of hysterical laughing which continued uninterruptedly for at least an hour. From this condition she passed for a few minutes into general muscular twitching. The velum palati, however, remained then, as well as until now, perfectly quiescent.

ETIOLOGY.—The choreiform movements of the soft palate, as they were seen in this case, doubtless had for their origin a reflex cause, situated in a neoplastic and hyperæsthetic condition of the intra-nasal mucous membrane. In view of the fact that another exciting element has been mentioned as figuring in the etiology of the affection, the nasal disease may be termed the remote exciting cause.

Reference to a quotation from Dr. Williams informs us that the peculiar disorder of the velum palati became apparent soon after the patient, when still a child, had experienced on two consecutive occasions severe fright. This, he maintains, developed by its immediate exciting influence the movements, which occurrence, it is my belief, simply brought into conspicuous activity what the reflex cause years before already had established.

The family history unmistakably illustrates a neurotic diathesis which evidently forms the predisposing cause.

Dr. C. E. Riggs, of this city, Professor of Mental and Nervous Diseases in the Medical Department of the University of Minnesota, who at one time saw the patient, personally related to me the following opinion when speaking of the nervous origin of the trouble:

"The antecedents and personal history of Miss Z., as related to me, clearly indicate the unstable and illy balanced condition which characterizes the nervous diathesis. Following the teaching of Pierret, we may regard all the intra-cranial nerves as analogous to a spinal nerve—the posterior root of this hypothetical nerve being the sensory portion of the fifth, its anterior root all the intra-cranial motor nerves, including the motor portion of the fifth. This hypothesis readily explains the clonic spasm of the levator muscles.

Catarrhal states of the mucous membrane, more especially of the nasal and pharyngeal passages, may have their origin in a poorly-nourished nervous system.

The facial nerve gives off in the aqueductus fallopii three branches, two of which, the great and small superficial petrosal nerves, furnish motor influence to the levator palati, the azygos uvulæ, the tensor palati and tensor tympani muscles; through Meckel's ganglion the great superficial petrosal nerve is distributed to the levators palati.

The diseased surfaces under consideration derive their sensation from the fifth. The catarrhal condition of these mucous surfaces may be considered both as a cause and an effect; but the prime factor in the evolution of this peculiar clonic spasm I believe to be the intense nerve sensitiveness of the patient, since without it there would have been no reflex excitation, but a purely local condition.

The excessive hyperæsthesia of the diseased tract clearly shows great irritation of the terminal sensory filaments of the fifth nerve.

Accepting Pierret's hypothesis that the cranial nerves, as a whole, comport themselves like a spinal nerve, the reflex nature of the spasm is readily describable."

That this is a rare case is quite evident; whether another just like it has as yet been reported is doubtful.

The cases reported by Sajous (Universal Annual) and by Burnett (Transactions of the American Otological Society, Vol. 3, Part 3) were, it appears, particularly associated with diseases of the eustachian tubes or ears, and the crackling noises characterizing them the authors ascribe to the aural complications. Mackenzie, in Wood's Reference Hand-book, Vol. 5, speaks of "Clonic spasm of the soft palate, with objective noises in the ear, dependent upon neuralgia of the trigeminus, have since been observed by Scheck."

A series of interrogations always gave negative results in the case I have just reported; no evidence of signs could be elicited which led to the belief that eustachian or aural disease had at any time existed.

Then as to the clicking sound: how was it produced? The levator muscles of the palate were the only ones engaged in the spasmodic actions; therefore, it is a natural conclusion that the click resulted from the combined effect of muscular rigidity and sudden disengagement of the velum palati from the pharyngeal vault.

